# Myogenic Potential of Extracellular Matrix Derived from Decellularized Bovine Pericardium

**DOI:** 10.3390/ijms22179406

**Published:** 2021-08-30

**Authors:** Flavia Carton, Dalila Di Francesco, Luca Fusaro, Emma Zanella, Claudio Apostolo, Francesca Oltolina, Diego Cotella, Maria Prat, Francesca Boccafoschi

**Affiliations:** 1 Department of Health Sciences, University of Piemonte Orientale “A. Avogadro”, 28100 Novara, Italy; flavia.carton@uniupo.it (F.C.); 20019023@studenti.uniupo.it (D.D.F.); 20029539@studenti.uniupo.it (E.Z.); 20014389@studenti.uniupo.it (C.A.); francesca.oltolina@med.uniupo.it (F.O.); diego.cotella@med.uniupo.it (D.C.); maria.prat@med.uniupo.it (M.P.); 2 Tissuegraft Srl, 28100 Novara, Italy; fusaro@tissuegraft.it

**Keywords:** tissue engineering, decellularized pericardium, extracellular matrix, skeletal muscle, myogenic differentiation

## Abstract

Skeletal muscles represent 40% of body mass and its native regenerative capacity can be permanently lost after a traumatic injury, congenital diseases, or tumor ablation. The absence of physiological regeneration can hinder muscle repair preventing normal muscle tissue functions. To date, tissue engineering (TE) represents one promising option for treating muscle injuries and wasting. In particular, hydrogels derived from the decellularized extracellular matrix (dECM) are widely investigated in tissue engineering applications thanks to their essential role in guiding muscle regeneration. In this work, the myogenic potential of dECM substrate, obtained from decellularized bovine pericardium (Tissuegraft Srl), was evaluated in vitro using C2C12 murine muscle cells. To assess myotubes formation, the width, length, and fusion indexes were measured during the differentiation time course. Additionally, the ability of dECM to support myogenesis was assessed by measuring the expression of specific myogenic markers: α-smooth muscle actin (α-sma), myogenin, and myosin heavy chain (MHC). The results obtained suggest that the dECM niche was able to support and enhance the myogenic potential of C2C12 cells in comparison of those grown on a plastic standard surface. Thus, the use of extracellular matrix proteins, as biomaterial supports, could represent a promising therapeutic strategy for skeletal muscle tissue engineering.

## 1. Introduction

Skeletal muscle represents 30–40% of human body mass and plays an essential role in protecting internal organs, producing movement in response to nervous systems, and regulating metabolism and homeostasis. This tissue is made of elongated multinucleated syncytia cells, known as myofibers, formed by fusion of precursor cells (myoblasts) during the embryonic development. Although adult skeletal muscle represents a permanent tissue, it has the ability to regenerate lost tissue after a minor injury through the activation of different cell types (e.g., satellite cells, mesenchymal stem cells, interstitial cells, blood vessels, and others) [1,2]. Among these, satellite cells (SCs), the myogenic progenitor cells localized between the basal lamina and the muscle fibers membrane, represent the main players in skeletal muscle regeneration. In fact, following injuries SCs, which are normally quiescent during homeostasis, re-enter cell cycle as myoblasts and start to proliferate and differentiate into myotubes that fuse within the damaged fibers [1].

Despite the notable regenerative potential of skeletal muscle, in the case of large injuries or extreme damage this capability is limited, thus, reducing the functionality of the muscle tissue. Common causes leading to progressive loss of muscle mass can be: traumatic injuries, tumor ablation, metabolic disorders, or inherited genetic disease such as muscular dystrophies, amyotrophic lateral sclerosis, and pediatric Charcot–Marie-Tooth disease [3,4,5,6]. Up to now, different strategies have been proposed in clinic to restore muscle tissue loss. Among them, autologous muscle transfer represents one of the most current clinical options but it is limited by donor tissue availability, donor-host compatibility, and poor engraftment [7,8]. Other potential strategies are represented by physical and gene therapies, cell transplantation, growth factors implementation, and others [9,10,11]. However, none of these strategies provides a complete functional restoration of the muscle tissue, especially in case of chronic or severe skeletal muscle injuries [12].

To overcome these limitations, tissue engineering (TE) has been intensively investigated to enhance new muscle formation by creating a cellularized tissue-like scaffold. The elected biomaterial must be biocompatible, biodegradable, non-immunogenic, and have adequate chemical and mechanical properties able to enhance and support cellular migration and growth. In the contest of muscular application, in order to recreate the native skeletal muscle, the biomaterial should be capable of enhancing the formation of new muscle myofibers, which should then be integrated within the surrounding tissue [10,13,14]. In this regard, researchers have investigated several biological scaffolds among which hydrogels represent a promising therapeutic tool for skeletal muscle regeneration. These materials have a three-dimensional (3D) structure with the unique ability to absorb a high amount of water or biological fluids, giving the possibility to the cells and molecules to spread in mimicking the native soft tissue [15,16]. This 3D network can be composed of materials with different origins: biological, synthetic, or composite [17]. As they induce a limited inflammatory response, biological hydrogels are a popular choice. Many of them are commonly formed by component of the extracellular matrix (ECM). Previous works have already successfully screened the potential of hydrogels, made of a single or of a combination of ECM proteins (e.g., collagen, elastin, agarose, fibrin, fibronectin, and laminin) for muscle development, although they are not able to recapitulate the complex biochemical microenvironments of ECM [18]. In this regard, biological hydrogel can also be obtained directly through tissue and organ decellularization with the advantage of preserving the native composition including collagen, glycosaminoglycans (GAGs), growth factors, and other signaling molecules required for cell differentiation and tissue homeostasis [19]. Indeed, in the contest of muscle regeneration, the role of ECM in regulating cellular motor, proliferation, migration, and myogenesis is well recognized [20,21,22,23,24,25,26]. In detail, in response to muscle injury, ECM undergoes gradual remodeling with the components of the basal lamina being degraded by matrix metalloproteinase, while growth factors and signaling molecules interacting with satellite cells start to proliferate and differentiate into myoblasts. In this process, the presence of ECM is essential to allow the interactions between growth factors and skeletal muscle satellite cells that are the main players in muscle repair [27]. Following the activation of SCs, other components of ECM such as collagen, fibronectin, elastin, and proteoglycans are responsible to facilitate myoblasts elongation, alignment, and fusion [28].

Herein we investigated the myogenic potential of a decellularized extracellular matrix obtained from bovine pericardium (dECM), a safe biomaterial already used in clinical practice [29,30]. In detail, we evaluated the effects of dECM on C2C12 myoblasts differentiating to myotubes from morphological and molecular points of view. To determine if the dECM niche can act as a myoblasts mitogen, the morphological and molecular features of C2C12 myotubes were evaluated during the distinct differentiation phase. Our results demonstrate that the biochemical cues provided by dECM can better support and promote muscle differentiation in comparison with the traditional plastic ware.

## 2. Results

### 2.1. Myotubes Formation on dECM Coating

**Figure 1:**
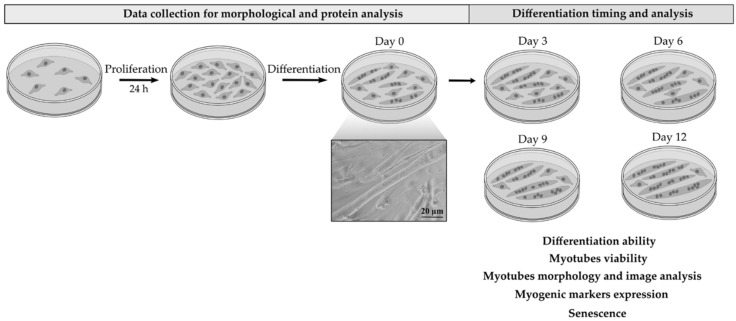
Temporal scheme of the treatments and of the analysis performed on C2C12 cells grown in the cell culture plates coated or not with dECM.

To verify that extracellular matrix prepared from the decellularized (dECM) bovine pericardium can be used as coating material to support myotubes formation, C2C12 myoblasts were cultured on a plastic surface coated with dECM at a concentration of 4 mg/mL. Cells were allowed to grow in a proliferation medium for about 24 h until they reached 80–90% confluence; following cells were cultured in differentiation medium containing 2% horse serum to promote their differentiation. The appearance of the first myotubes, approximately 2/3 days after cells were cultured in differentiation medium, was marked as day 0 and days were counted from this one and marked as 3, 6, 9, and 12 days. This time course approach was maintained for all the experiments (Figure 1). These cultures were thus compared with parallel cultures carried out on uncoated plastic plates (CTR), to evaluate the potential of dECM molecules in myotube formation.

**Figure 2:**
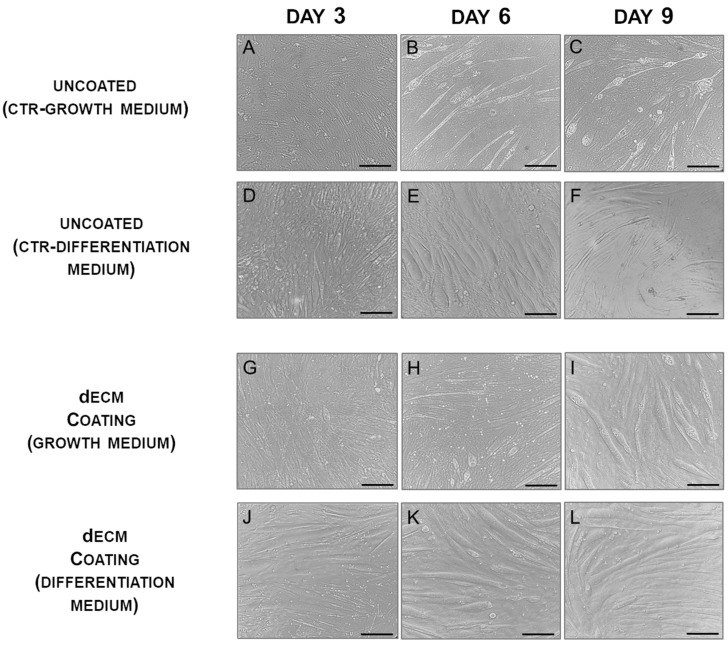
dECM enhances the differentiation of C2C12 myoblasts to myotubes. Cells were induced to differentiate in low serum medium in plates either uncoated (2 top rows) or coated (2 bottom rows) with dECM. Representative images of cultures at day 3 (**A**,**D**,**G**,**J**); at day 6 (**B**,**E**,**H**,**K**); at day 9 (**C**,**F**,**I**,**L**) post-differentiation. Scale bars: 100 µm.

C2C12 cells, when cultured on dECM coating under differentiation condition, were able to differentiate showing a typical myotube morphology, characterized by cells elongated containing three or more nuclei within a single membrane structure. Indeed, by day 3, the cells formed elongated and aligned structures, with few undifferentiated myoblasts. This pattern was maintained over the following days (Figure 2J–L). Furthermore, myoblasts grown on dECM with basic growth medium were still able to form multinucleated myotubes but cytoplasmic vacuolation was clearly visible on day 6 and was even more evident on day 9, indicating a non-optimized differentiation environment (Figure 2G–I). In contrasts, cultures performed on uncoated plastic plates in a differentiation medium showed irregularly distributed and sized myotubes (Figure 2D–F). To further evaluate the myogenic potential of dECM, C2C12 were left to differentiate in grown medium on plastic plates (Figure 2A–C). In these conditions myoblasts were still able to form multinucleated cells but a delayed cytoplasmic vacuolization, caused by a not optimized differentiative environment, was observed in myotubes grown on dECM compared to the CTR condition.

### 2.2. Myotubes Viability on dECM Coating

**Figure 3:**
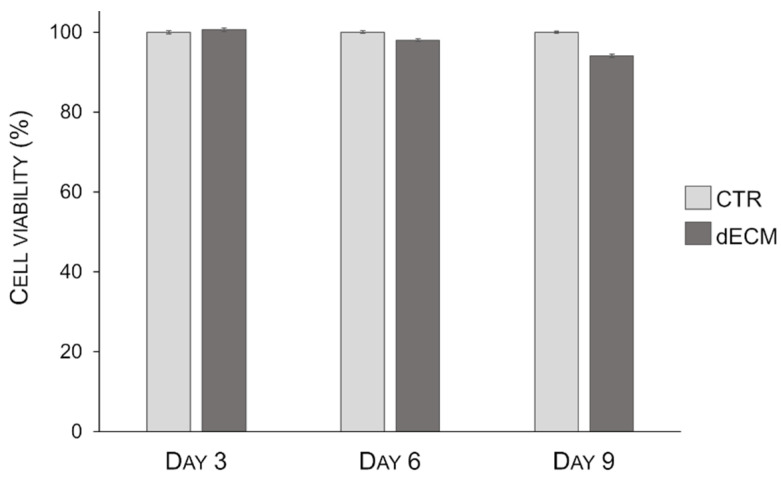
Effect of dECM coating on C2C12 viability. The cells were differentiated on plates either uncoated or coated with dECM up to 9 days and viability was measured by MTT assay. Cell viability is referred to C2C12 cells grown directly on plastic, which is taken as 100% viability. The graphic shows the mean value ± S.E. of percentage of cell viability observed in three independent experiments carried out in triplicates. Statistic was determined by *t*-test.

To verify the dECM citocompatibility and the role of dECM proteins on cells viability, a MTT assay was performed after 3, 6, and 9 days post differentiation. The results show that dECM was able to support cells viability up to 9 days, the longest test timing, without any statistically significant decrease in cell viability with respect to control (Figure 3).

### 2.3. Myotubes Morphology and Fusion Index

**Figure 4:**
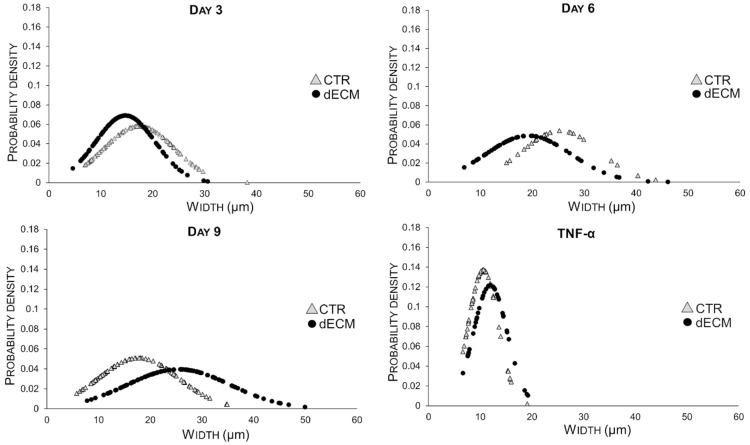
Time course of morphological changes (width) of C2C12 derived myotubes grown for 3, 6, and 9 days on plastic plates either uncoated or coated with dECM. The data are expressed as Gaussian curves with *n* ≥ 70. Measures were taken from bright filed images (20× magnification).

The morphology of the differentiated myotubes was examined in detail, i.e., by measuring width and length in different culture conditions and at different times on the basis of the bright field images. As shown in Figure 4, in the case of cultures plated on dECM coated substrates the width of myotubes increased constantly over the time until 9 days. By contrast, myoblasts plated on plastic substrate (CTR) gave rise to myotubes significantly wider than those differentiated on dECM up to day 6, but at day 9 their width decreased significantly. In both cases the normal distribution of the Gaussian curves became larger with time, suggesting an increased variability in size.

**Figure 5:**
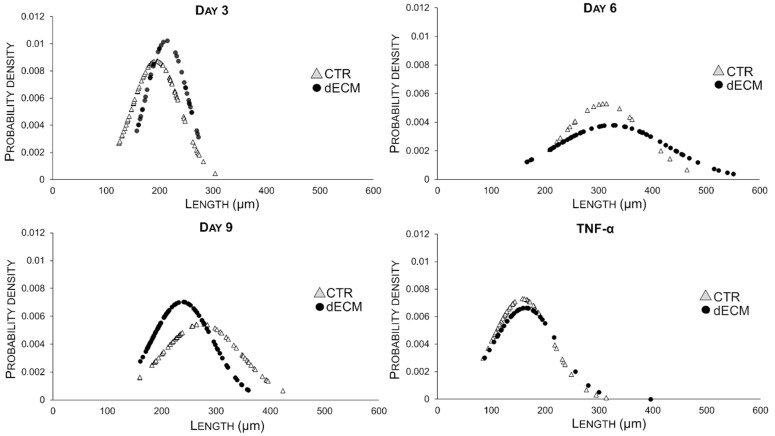
Time course of morphological changes (length) of C2C12 derived myotubes grown for 3, 6, and 9 days on plastic plates either uncoated or coated with dECM. The data are expressed as Gaussian curves with *n* ≥ 70. Measures were taken from bright filed images (20× magnification).

On the other hand, the length of myotubes grown on dECM increased significantly at 3 and 6 days, suggesting a rapid fusion between the single cells to form the first multinucleated myotubes (Figure 5). At day 9, however, the length of these myotubes decreased compared to that of myotubes obtained in the absence of dECM.

**Figure 6:**
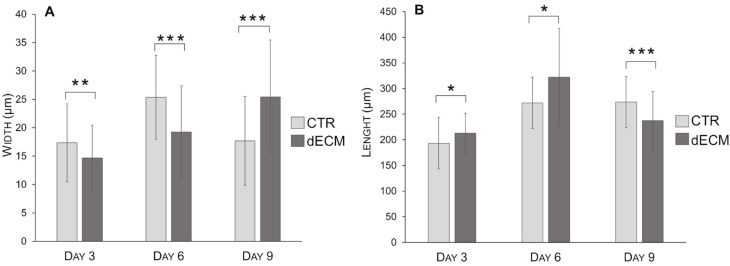
Morphological features of C2C12 myotubes grown on plastic plates either uncoated (CTR) or coated with dECM for different periods. Width (**A**) and length (**B**) are reported. Statistic was determined by *t*-test. * *p* < 0.05, ** *p* < 0.01, *** *p* < 0.001. A minimum of 70 myotubes were measured in each condition or time.

Thus, in the case of cells grown on dECM the secondary fusions were more involved in increasing the width than the length of myotubes, as summarized in Table 1 and Figure 6. In both measurements (width and length) myotubes treated with 20 ng/mL of TNF-α, a hallmarks of muscle atrophy, were used as negative control (Figure 4 and Figure 5). The data of these morphological analysis are summarized in Table 1 and in the graph of Figure 6.

**Figure 7:**
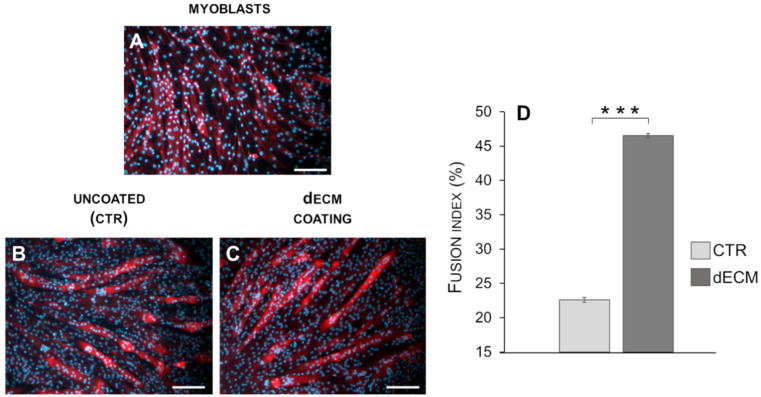
Fluorescence analysis of myotubes induced to differentiate in low serum medium in plates either uncoated ((**B**): CTR) or coated (**C**) with dECM. Cells at day 9 post differentiation were stained with phalloidin (red fluorescence) to highlight cytoplasm and with DAPI (blue fluorescence) to depict nuclei. Undifferentiated myoblasts were used as control (**A**). dECM coating induces fusion of C2C12 myotubes, as shown by the fusion index taken 9 days post-differentiation (**D**). Statistic was determined by *t*-test, *** *p* < 0.001. Scale bars: 200 µm.

The extent of differentiation of C2C12 cells was measured also by fusion index, which reflects multinucleated myotubes formation [31,32]. Fusion index was calculated as the average number of nuclei contained in myotubes, compared with the total number of nuclei. Differentiation index of C2C12 myoblasts was found to be significantly increased in cells grown for 9 days on the substrates coated with dECM with respect to controls (Figure 7).

### 2.4. Myogenic Differentiation of dECM Coating

To confirm the observation that dECM activates a differentiation program in skeletal myoblasts, we have verified the ability of dECM to induce the expression of α-sma, myogenin and MHC proteins, as detected by Western blot. While MHC is a late differentiation marker, myogenin is a helix-loop-helix transcription factor whose expression is induced early in differentiation, preceding cell cycle exit; finally α-sma is a myogenic marker normally produced by the cells few hours after the exposition to differentiation medium, which is then lost with the onset of contractility phenotype [33]. C2C12 cells were cultured either on uncoated or dECM pre-coated plastic wells and 3, 6, 9, and 12 days post-differentiation and the expression of early and late myogenic differentiation markers was measured by Western blot of whole cell lysates, and the intensity of the bands was quantified.

**Figure 8:**
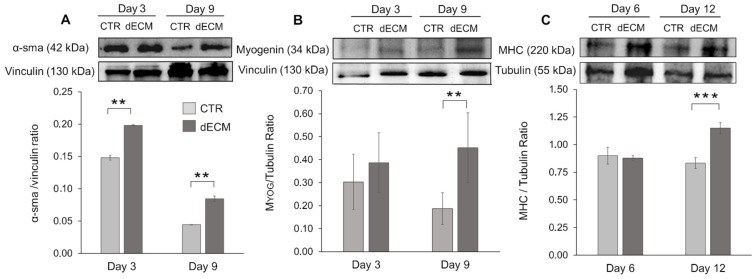
Expression of myogenic markers, analyzed by Western blot. C2C12 myoblasts were incubated either on uncoated (CTR) or dECM pre-coated wells and induced to differentiate in differentiation medium. At different time points the contents of α-smooth muscle actin (**A**), myogenin (**B**), and MHC (**C**) were measured by Western blots of whole cell lysates and the intensity of the bands quantified, relative to the housekeeping proteins vinculin or tubulin used to normalize. Representative Western blots out of the three for each condition are shown. Statistics were determined by *t*-test. ** *p* < 0.01, *** *p* < 0.001.

Figure 8A shows that the expression of α-sma was significantly increased at 3 and 9 days in the cells cultured onto dECM in comparison to control cells grown directly on the uncoated substrate. In both cases α-sma expression was decreased at day 9, as expected for an early differentiation marker.

By contrast, the early gene myogenin was found to be expressed at significantly higher levels in myotubes cultured on dECM at 9 days post-differentiation, while in the case of myotubes differentiated on plastic surfaces its expression was down-modulated with time (Figure 8B).

Finally, the expression of the terminal differentiation marker MHC was significantly increased at 12 days in myotubes grown onto dECM in comparison to control cells (CTR) grown in the absence of dECM, where the expression of MHC was stable over time (Figure 8C). These results confirm that the dECM is able to promote and enhance both early and late steps of skeletal muscle differentiation.

### 2.5. Senescence Evaluation

**Figure 9:**
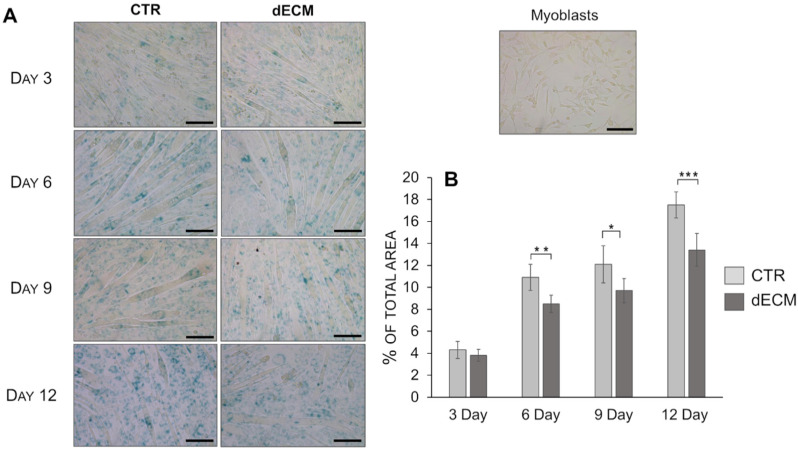
Representative images (**A**) of myotubes grown either on uncoated (CTR) or dECM pre-coated substrates up to 12 days and stained for the senescence-associated β-galacotosidase (SA-βGal) marker. Quantification of SA-βGal positive cells (**B**) expressed as percentage of total area (*n* = 6). * *p* < 0.05, ** *p* < 0.01, *** *p* < 0.001. Scale bars: 100 µm.

To evaluate the ability of dECM to support and maintain myotubes proliferation, the senescent potential was evaluated by staining the cells with SA-β-Gal, which is a specific biomarker for replicative senescence [34]. Indeed, the number of myotubes stained in blue was higher on plastic substrate with respect to the ones detectable in cultures on dECM starting from day 6 (Figure 9). Moreover, after 12 days post-differentiation, the multinucleated myotubes formed on CTR seem to be thinner than those formed on dECM substrate.

## 3. Discussion

The current need to provide therapeutic strategies to repair muscle injuries and wasting paved the way for tissue engineering applications. In particular, hydrogels biomaterials represent one possible option for skeletal muscle regeneration [17,18]. Among them, natural hydrogels derived from extracellular matrix (ECM) are an attractive biomaterials thanks to the essential contribute provided by ECM micro-environment during skeletal muscle development and repair [28]. For this purpose, an effective decellularization protocol must be applied to remove all the cellular components that may evoke immunogenicity without disrupting the native ECM structure necessary for a good cell–cell and cell–ECM interactions. In this study the myogenic potential of extracellular matrix components derived from bovine pericardium (dECM) was demonstrated in vitro on C2C12 myoblast cells.

The non-toxicity of the decellularized ECM matrix was assessed by evaluating myotubes viability compared to the plastic standard growth surface. The results obtained revealed that both the decellularization experimental procedure and bovine pericardium extract does not contain factors that could inhibit C2C12 grown as already observed for other cell lines by Li et al. [35]. Once proved the adequate biocompatibility of decellularized bovine pericardium, the myogenic potential of dECM matrix was evaluated. The development of skeletal muscle represents a complex multistep process that occur both during embryogenic development and in postnatal life in the case of muscle injuries. This process is characterized by specific spatiotemporal events that are strictly controlled by signaling molecules and transcription factors with many of them specific during muscle myogenesis [36]. The comprehension of these mechanisms represents an acute need to develop new therapies in the field of muscle disease and regeneration (e.g., volumetric muscle loss (VLM), muscle dystrophies, neuromuscular disease, and sarcopenia). In particular, muscle differentiation is divided into several distinct phase. The first stages are characterized by migration and proliferation of myoblasts followed by proliferative arrest in which the cells escape from cell cycle in favor of migration and fusion. To obtain this, the reduction of serum concentration in a culture medium is commonly applied for in vitro studies. In the following step, myoblasts start to fuse in two distinct phase processes: the primary fusion, in which the single myoblasts fuse each other’s forming an initial multinucleated cell, and the secondary fusion where other myoblasts interact with the neo-formed myotubes leading to the formation of mature myotubes characterized by high cytoplasmic volume and size [37]. All these processes are tightly controlled by transcription and myogenic factors regulated by extracellular signals such as growth factors, ECM, and cell–cell interactions [38]. Among the different myogenic signaling mechanisms, the cell–ECM relationships between the basal lamina and endomysium, largely mediated by integrins, play an essential role in regulating muscle development and physiology [26,39].

The decellularization protocol of bovine pericardium applied in this study allowed the preservation of the main ECM components such as collagens, glycosaminoglycans (GAGs), elastin and basement membrane molecules (e.g., laminin and collagen IV), which could eventually provide chemical signals for differentiation.

Among these molecules, laminin and collagen IV are known to play an essential role during cell adhesion and migration [40]. Indeed, previous studies have shown that surfaces coated with laminin act as myoblasts mitogen by increasing cell adhesion and migration compared to surface coated with others ECM proteins such as fibronectin, type I and IV collagen [41,42]. The importance of basal membrane integrity after decellularization process in maintaining cell-matrix interactions have been also proved by Xing Q. and collaborators. Indeed, they demonstrated an increased expression of integrin (α_3_β_1_ and α_11_β_1_) by human mesenchymal stem cells (hMSCs) grown on bovine ECM niche, thus, confirming their essential role in mediating cell adhesion and migration [43]. In the contest of muscle development, similar results were obtained by Ding et al. that proved the enhanced expression of integrin α_7_β_11,_ commonly present in myoblasts, on C2C12 cells grown on Matrigel [44]. Thanks to this intensified expression, the interactions between integrin and extracellular proteins, such as tenascin-C and Nephronectin, increased accelerating the myogenic processes [45,46]. Laminin can influence myoblasts differentiation also indirectly by changing cell–cell affinity that represents another crucial step during myogenesis [37,41]. Moreover, previous studies demonstrated that other specific cell proteins involved in cell–cell recognition such as N- and M-cadherin appeared to be altered on ECM substrates such as Matrigel [39,44].

Although the laminin directs muscle formation, other ECM components such as collagen and proteoglycan seem to have an important role in regulating myogenesis. In particular, collagen type I, that represents the main component of decellularized bovine pericardium, has an essential role in both morphological and biochemical differentiation [25,26,47]. Collagen type VI seems to have an essential role on the regulation of satellite cell self-removal, while fibronectin contributes to myoblasts adhesion, migration, and differentiation [48,49]. Finally, proteoglycans, such as decorin and perlecan, are involved in myogenic processes through the modulation of growth factors activities (fibroblast growth factor 2 (FGF-2) and hepatocyte growth factor (HGF)) commonly involved in muscle development [50,51,52].

The role of dECM components in facilitating the different phase of myogenesis was also confirmed in this work. In particular, microscope images and morphological analysis (length) showed that the presence of peptide and proteins, derived from dECM niche, was able to enhance myoblasts fusion into the first multinucleated myotubes already after 3 and 6 days post differentiation. In fact, at this time, more extensive myotubes with fewer mononucleated cells were observed on dECM coating followed by a smaller number of roundish cells visible in CTR conditions. From day 9 forward, the values of length of myotubes grown on dECM, significantly decreased compared to CTR indicating that, at this point, the secondary fusion characterized by the increase of cytoplasmic volume and size prevails over the length growth. In fact, at day 9, both myotubes width and fusion index parameters increased on dECM substrate, suggesting that at this stage of differentiation the fusion of new myoblasts within the neo-formed myotubes was facilitated by the biochemical stimuli provided by dECM. Finally, the myogenic potential of dECM environments was also confirmed by the expression of specific molecular markers. Indeed, cells grown on dECM expressed nearly always significantly higher levels of the three markers analyzed, i.e., α-sma, myogenin and MHC, in comparison to cells grown directly on plastic substrates. As expected α-sma was down-regulated at day 9 in both culture conditions since it is encoded by a very early differentiation gene [33]. Finally, in cells grown on dECM both myogenin, encoded by a relatively early gene and MHC, a marker of advanced maturation of skeletal muscle, were expressed at higher levels than in cells grown on plastic substrate in the latest time point examined (9 and 12 days, respectively). These data are in line with previous reports in which different natural hydrogels (e.g., gelatine or ECM derived from skeletal muscle) were reported to enhance the proliferation and differentiation of myogenic cells, including C2C12 myoblasts [42,53,54,55].

Finally, in the contest of muscle repair, the regenerative capacity is strictly related to the activation, proliferation and differentiation of satellite cells. Among them, it is well known that the proliferative ability of human myoblasts is compromised by replicative senescence; a terminal growth arrest characterized by a progressive telomerase shortening [56]. In this study, the ability of extracellular matrix proteins to maintain and support skeletal muscle development was also confirmed by senescence analysis. Indeed, the replicative senesce was delayed in myotubes grown on dECM surface underling the important role played by matrix molecules and growth factors on proliferative capacities.

## 4. Materials and Methods

### 4.1. Decellularization of the Bovine Pericardial Extracellular Matrix

Natural hydrogel obtained from the decellularized bovine pericardium was kindly provided by Tissuegraft Srl (Novara, Italy) (patent number 102020000007567 submitted on 9 April 2020). The efficiency of decellularization process, while preserving the total matrix proteins was evaluated and confirmed by Tissuegraft Srl. Briefly, the hydrolyzed extracellular matrix was obtained by decellularized bovine pericardium, after being digested and lyophilized. The standardized process guarantees to preserve the main components of the extracellular matrix mainly composed of 90% collagen, 5% elastin, and 1% glycosaminoglycans among which 0.45% was hyaluronic acid, while efficiently eliminating the cellular component.

### 4.2. Cell Cultures

The decellularized extracellular matrix was used for coating (dECM) at a final concentration of 4 mg/mL. The diluted dECM was added to the well plates and left for 1 h at 4 °C. Excess substrate was aspirated, and the plates were rinsed with PBS before cell seeding.

C2C12 myoblasts (cell line CRL-1772 from ATCC) were plated onto pre-coated flat-bottom well plates and maintained under a humidified atmosphere with 5% CO_2_ at 37 °C, in Dulbecco’s modified Eagle medium (DMEM—Euroclone, Milan, Italy), supplemented with 10% (*v*/*v*) fetal bovine serum (FBS), penicillin (100U/mL), streptomycin (0.1 mg/mL), amphotericin (0.25 ug/mL) and l-Glutamine (2 mM) (all products from Euroclone, Milan, Italy). When myoblasts reached a 70–80% confluence, they were cultured both in growth and differentiation medium containing 2% horse serum (Euroclone, Milan, Italy) and left to differentiate up to 12 days, changing the medium every 2/3 days. All the experiments were performed following the specific time course mentioned below. Cells plated directly on plasticware (CTR) were used as control.

### 4.3. Cells Morphology

Cells were seeded on glass coverslips in 24 multiwell plates at a density of 18.000 cells/cm^2^ and, once they reached the confluence, about 24 h post plating, they were switched to differentiation medium and were cultured for 3, 6, 9, and 12 days. For bright field, qualitative images were obtained directly from 24 multiwell plates using IM-3 OPTIKA inverted microscope equipped with a digital microscope camera (Leica DFC320, Wetzlar, Germany). For fluorescence microscopy, cells were fixed with 4% formalin in phosphate buffered saline (PBS) pH 7.4 for 1h at room temperature, rinsed with PBS, permeabilized with 0.5% Triton X-100 in PBS, and incubated with Phalloidin–Tetramethylrhodamine B isothiocyanate (TRITC), diluted 1:1000 (Sigma Aldrich, Milan, Italy) for 45 min at 37 °C. Cell nuclei were stained with 300 nM Diamidine-20-phenylindole dihydrochloride (DAPI, Sigma Aldrich, Milan, Italy) for 2 min. After rinsing, samples were processed in mounting medium (60% glycerol in PBS) and observed under a fluorescence microscope (DFC7000 T Leica, Wetzlar, Germany). Each treatment was performed in triplicate, and each experiment was repeated at least two times. Images were acquired (10 field/well) and analyzed to determine differentiation and fusion indexes. Representative images were taken for each condition and analyzed to evaluate myotube formation.

### 4.4. Cell Viability Assay

C2C12 myoblasts were seeded onto either uncoated or dECM-coated flat-bottom 96 multiwell plates at the density of 18.000 cells/cm^2^. When confluence was reached, cells were incubated in differentiation media for different time points (3, 6, and 9 days). At each time point, cells were treated with MTT (Sigma Aldrich, Milan, Italy) stock solution (5 mg/mL,) at one tenth of the original culture volume and incubated for 4 h. Then, formazan crystals, produced by mitochondrial activity, were dissolved in 100 µL of dimethyl sulfoxide (DMSO, Sigma-Aldrich, Milan, Italy) and the optical density was measured in a multiwell reader (2030 Multilabel Reader Victor TM X4, PerkinElmer, Milan, Italy) at 570 nm. Viability of cells cultured on CTR were taken as 100% viability, and values obtained from cells undergoing the different treatments were referred to this value. Experiments were performed at least for 3 times using 3 replicates for each sample.

### 4.5. Morphometry and Image Analysis

Morphometric analyses of myotubes were carried out by measuring cell width, length, and fusion index (FI) using ImageJ software 1.52r (ImageJ, USA). Cells were seeded on glass coverslips in 24 multiwell plates either uncoated or pre-coated with dECM at the density of 18.000 cells/cm^2^ and images were acquired both in phase-contrast (IM-3 OPTIKA microscope; 20×) for diameter and length evaluation and in fluorescence (DM2500 Leica microscope; 10×) for FI analysis. At least fifteen fields were randomly collected for each condition (dECM or CTR) and a minimum of 70 myotubes were evaluated. In particular, the width is reported as the average of three different values derived from multiple measurements for myotubes made along the length, as already published [57,58]. Myotube lengths were obtained by measuring the longitudinal axis of the myotubes that were not longer than the field of view. As negative control cells were treated with tumor necrosis factor-α (20 ng/mL) (TNFα Sigma Aldrich, Milan, Italy) for 72 h. From these values, Gaussian functions, used to represent the probability density, were obtained. Probability density provided, in this case, a graphical representation of the morphology of myotubes in a heterogeneous spatial contest. In detail, this type of representation describes real values that tend to concentrate around an average value. When a phenomenon X has a normal distribution the graph assumes a typical “bell shape” and the value in the center represent the highest probability of occurring while on both sides of the curve the probability decreases. To obtain the probability density, the length and width of at least 70 myotubes were measured using the ImageJ software. Then, the normal distribution of each measure was calculated in excel applying the following formula: = NORMAL.DISTRIB.N (MEASURE; MEAN of all measurements performed; DEV.ST; FALSE).

For fusion index (FI) analysis, the total number of nuclei was determined by DAPI-positive counting (10× magnification). Myogenic index was calculated by dividing the number of nuclei present in myotubes by the total number of nuclei in the whole field. Results were expressed as the mean ± SD of FI. All the images were captured using a Leica DFC320 (Wetzlar, Germany) camera for inverted microscope and DM2500 Leica, (Wetzlar, Germany) for fluorescence.

### 4.6. Determination of Myogenic Differentiation by Western Blot

Myoblasts were seeded onto dECM pre-coated 6 well plates at a cell’s density of 7.000 cells/cm^2^. Once confluence was reached cells were switched to differentiation media and cultured for 3, 6, 9, and 12 days. At the end of each time point cells were lysed in RIPA buffer (50 mM Hepes pH 7.4, 150 mM NaCl, 0.1% SDS, 1% TX-100, 1% DOC, 10% Glycerol, 1.5 mM MgCl_2_, 1mM EGTA, 1 mM NaF) supplemented with 100 mM phenylmethylsulfonyl fluoride (PMSF), and a cocktail of protease inhibitors (Sigma-Aldrich, Milan, Italy). The total protein concentration was determined using bicinchoninic acid assay (Pierce, Waltham, IL, USA) and equal amounts of proteins (50 µg) in Laemmli buffer (62.5 mM Tris-HCl pH 6.8, 10% glycerol, 5% β-mercaptoethanol, 0.005% bromophenol blue, 2% SDS; Sigma Aldrich, Milan, Italy) were separated by SDS-polyacrylamide gel electrophoresis (SDS-PAGE) and transferred to nitrocellulose membranes (Amersham Biosciences, Little Chalfont, UK). The membranes were blocked with 5% dry milk and incubated overnight at 4 °C with the following antibodies; rabbit anti α-smooth muscle actin (anti α-SMA, 1:500; Abcam, Cambridge, UK), mouse anti-myogenin (1:500; Abcam, Cambridge, UK) and mouse anti-myosin heavy chain (anti-MHC, 1:1000; Merck Millipore, Milan, Italy). They were then washed and the expected proteins were revealed with the appropriate peroxidase conjugated secondary antibodies—(1:2000; Perkin-Elmer, Milan, Italy). The bands were acquired using a chemosensitive visualizer (ChemiDoc™ Touch Imaging System, Bio-Rad, Milan, Italy) and the intensity was measured with ImageLab software. Results were normalized with the housekeeping proteins vinculin and tubulin (anti-vinculin, anti-tubulin 1:1000; Merck Millipore, Milan, Italy).

### 4.7. Senescence Assay

The expression of β-galactosidase (SA-β-Gal) activity, a known characteristic of senescent cells, was measured following the instructions of Senescence Detection Kit (Abcam’s, Cambridge, UK). Briefly, cells were seeded in a 12-well plate (4.000 cells/cm^2^) and let to differentiate into myotubes for 3, 6, 9, and 12 days. The images of SA-β-Gal positive cells were collected using a phase contrast microscopy (20× magnification) and captured with a digital camera (Leica MC 120 HD, Wetzlar, Germany). Quantification of SA-β-Gal-positive cells was performed evaluating 6 stained areas chosen randomly for each condition and expressing the percentage of positive cells areas with respect to the total area using ImageJ software.

### 4.8. Statistical Analysis

All data were expressed as mean values ± standard deviation. Student’s *t*-test was used for evaluating the significance of the results obtained. *p*-value was calculated and the differences between variables with a value of *p* < 0.05 were considered statistically significant (* *p* < 0.05, ** *p* < 0.01, *** *p* < 0.001). Each experiment was repeated three times.

## 5. Conclusions

In this work, we focused on the in vitro effects of dECM components, derived from decellularized bovine pericardium, on the differentiation and maturation of myogenic cells. Our results suggest that such dECM positively influences myotube formation by providing the structural support and biochemical cues required during muscle development. In fact, the results obtained confirmed that this matrix supports the formation of myotubes, adequately expressing the contractile phenotype for early and late markers. Furthermore, differentiated C2C12 grown on dECM significantly delays the senescence process. These data indicate a possible use of this matrix in the regeneration of muscle tissue, but further experiments are needed to better characterize the role of dECM in regenerative processes. In conclusion, this relatively simple and easy substrate, which can be supplied as a by-product of the food industry allowing to participate in the modern concept of circular economy, could represent an interesting biomaterial useful for overcoming the problems related to muscle injuries or diseases improving patient quality of life.

## Figures and Tables

**Table 1:** Comparison of the morphological features (width, left and length, right) of myotubes originated from C2C12 cells grown on plastic plates either uncoated or coated with dECM. Values are expressed in micrometers mean ± SD and were obtained from a minimum of 70 myotubes. * *p* < 0.05, ** *p* < 0.01, *** *p* < 0.001.

Myotubes Width (µm)	CTR	dECM	*p* Values	Myotubes Length (µm)	CTR	dECM	*p* Values
Day 3	17.30 ± 6.86	14.67 ± 5.77	**	Day 3	193.45 ± 45.54	213.13 ± 38.90	*
Day 6	25.39 ± 7.39	19.23 ± 8.19	***	Day 6	310.84 ± 75.27	324.34 ±105.31	***
Day 9	17.70 ± 7.81	25.43 ± 10.04	***	Day 9	273.57 ± 73.16	237.83 ± 56.70	***

## Data Availability

Not applicable.
